# Supplementary figures and images for: The health benefits of volunteering at a free, weekly, 5 km event in the UK: A cross-sectional study of volunteers at parkrun
Source: PLOS Glob Public Health. 2022 Feb 24;2(2):e0000138. doi: 10.1371/journal.pgph.0000138 (PMC10021615; doi:10.1371/journal.pgph.0000138)

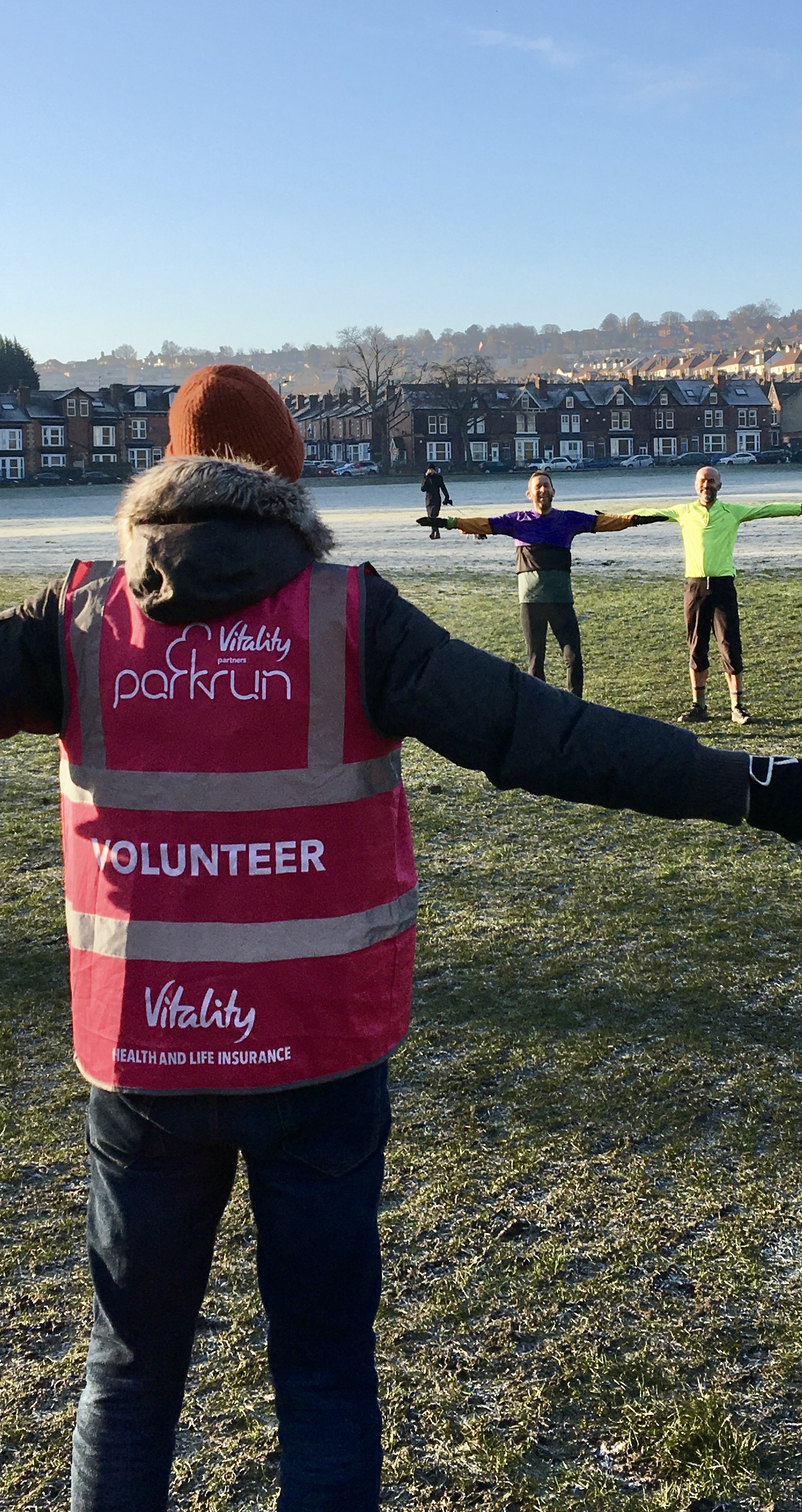

Supplement: S1 Fig — (JPEG) [file pgph.0000138.s001.jpeg]
