# Supplementary material for: The health benefits of volunteering at a free, weekly, 5 km event in the UK: A cross-sectional study of volunteers at parkrun
Source: PLOS Glob Public Health. 2022 Feb 24;2(2):e0000138. doi: 10.1371/journal.pgph.0000138 (PMC10021615; doi:10.1371/journal.pgph.0000138)
Supplement: S2 File — (DOCX) [file pgph.0000138.s003.docx]

S2 File. Analysis of walking and running instances in volunteers

| Runs | Count in 2018 | Cumulative count in 2018 | Cumulative proportion in 2018 |
| --- | --- | --- | --- |
| 0 | 110 | 110 | 48.5% |
| 1 | 39 | 149 | 65.6% |
| 2 | 15 | 164 | 72.2% |
| 3 | 5 | 169 | 74.4% |
| 4 | 5 | 174 | 76.7% |
| 5 | 8 | 182 | 80.2% |
| 6 | 10 | 192 | 84.6% |
| 7 | 3 | 195 | 85.9% |
| 8 | 2 | 197 | 86.8% |
| 9 | 2 | 199 | 87.7% |
| 10 | 5 | 204 | 89.9% |
| 11 | 4 | 208 | 91.6% |
| 12 | 0 | 208 | 91.6% |
| 13 | 3 | 211 | 93.0% |
| 14 | 2 | 213 | 93.8% |
| 15 | 6 | 219 | 96.5% |
| 16 | 0 | 219 | 96.5% |
| 17 | 1 | 220 | 96.9% |
| 18 | 0 | 220 | 96.9% |
| 19 | 1 | 221 | 97.4% |
| 20 | 0 | 221 | 97.4% |
| 21 | 0 | 221 | 97.4% |
| 22 | 0 | 221 | 97.4% |
| 23 | 2 | 223 | 98.2% |
| 24 | 1 | 224 | 98.7% |
| 25 | 0 | 224 | 98.7% |
| 26 | 0 | 224 | 98.7% |
| 27 | 1 | 225 | 99.1% |
| 28 | 0 | 225 | 99.1% |
| 29 | 0 | 225 | 99.1% |
| 30 | 0 | 225 | 99.1% |
| 31 | 0 | 225 | 99.1% |
| 32 | 0 | 225 | 99.1% |
| 33 | 1 | 226 | 99.6% |
| 34 | 0 | 226 | 99.6% |
| 35 | 0 | 226 | 99.6% |
| 36 | 0 | 226 | 99.6% |
| 37 | 0 | 226 | 99.6% |
| 38 | 0 | 226 | 99.6% |
| 39 | 0 | 226 | 99.6% |
| 40 | 0 | 226 | 99.6% |
| 41 | 1 | 227 | 100.0% |
