# Supplementary material for: The health benefits of volunteering at a free, weekly, 5 km event in the UK: A cross-sectional study of volunteers at parkrun
Source: PLOS Glob Public Health. 2022 Feb 24;2(2):e0000138. doi: 10.1371/journal.pgph.0000138 (PMC10021615; doi:10.1371/journal.pgph.0000138)
Supplement: Attachment — Submitted filename: Response to reviewers.docx [file pgph.0000138.s004.docx]

PGPH-D-21-00850
The health benefits of volunteering at a free, weekly, 5 km event in the UK: a cross-sectional study of volunteers at *parkrun
PLOS Global Public Health

Dear Dr. Haake,

Thank you for submitting your manuscript to PLOS Global Public Health. After careful consideration, we feel that it has merit and some minor changes are needed to fully meet PLOS Global Public Health’s publication criteria. Therefore, we invite you to submit a revised version of the manuscript that addresses the points raised during the review process.*

*Please submit your revised manuscript by Jan 07 2022 11:59PM. If you will need more time than this to complete your revisions, please reply to this message or contact the journal office at* [*globalpubhealth@plos.org*](mailto:globalpubhealth@plos.org)*. When you're ready to submit your revision, log on to* [*https://www.editorialmanager.com/pgph/*](https://www.editorialmanager.com/pgph/) *and select the 'Submissions Needing Revision' folder to locate your manuscript file.

Please include the following items when submitting your revised manuscript:*

- *A rebuttal letter that responds to each point raised by the editor and reviewer(s). You should upload this letter as a separate file labeled 'Response to Reviewers'.*
- *A marked-up copy of your manuscript that highlights changes made to the original version. You should upload this as a separate file labeled 'Revised Manuscript with Track Changes'.*
- *An unmarked version of your revised paper without tracked changes. You should upload this as a separate file labeled 'Manuscript'.*

*Guidelines for resubmitting your figure files are available below the reviewer comments at the end of this letter.

We look forward to receiving your revised manuscript.

Kind regards,

Chunxiao Li, Ph.D.
Academic Editor
PLOS Global Public Health*

*Journal Requirements:*

1. Peer review at PLOS ONE is not double-blinded (<https://journals.plos.org/plosone/s/editorial-and-peer-review-process>). For this reason, authors should include in the revised manuscript all the information removed for blind review.

**The information on ethical approval has now been included as follows: “The original survey was approved by Sheffield Hallam University Research Ethics Committee (Reference number: ER7034346). Written informed consent was received from all participants via the first page of the online survey.”**

2. Please update the completed 'Competing Interests' statement, including any COIs declared by your co-authors. If you have no competing interests to declare, please state "The authors have declared that no competing interests exist". Otherwise please declare all competing interests beginning with the statement "I have read the journal's policy and the authors of this manuscript have the following competing interests:"

**"I have read the journal's policy and the authors of this manuscript have the following competing interests: SH is Chair and AB and HQ members of the parkrun Research Board, an independent body tasked with reviewing research applications to parkrun. All authors are parkrun registrants/participants.”**

3. We have noticed that you have uploaded supporting information but you have not included a list of legends.  Please add a full list of legends for all supporting information files (including figures, table and data files) after the references list.

**A full list of legends for figures, tables and data files has been inserted after the references. Apologies for the omission.**

4. In the online submission form, you indicated that "Datasets relating to this manuscript are stored in the Sheffield Hallam University Research Database (SHURDA). Access is allowed in accordance with the Data Protection Act 2018 and the General Data Protection Regulation 2018. A copy of the full sample of anonymised data used in the manuscript is a available to researchers through the parkrun Research Board as outlined in the participant information sheet, subject to ethics approval and a data sharing agreement. Contact the corresponding author for access details."

All PLOS journals now require all data underlying the findings described in their manuscript to be freely available to other researchers, either 1. In a public repository, 2. Within the manuscript itself, or 3. Uploaded as supplementary information. This policy applies to all data except where public deposition would breach compliance with the protocol approved by your research ethics board. If your data cannot be made publicly available for ethical or legal reasons (e.g., public availability would compromise patient privacy), please explain your reasons by return email and your exemption request will be escalated to the editor for approval. Your exemption request will be handled independently and will not hold up the peer review process, but will need to be resolved should your manuscript be accepted for publication. One of the Editorial team will then be in touch if there are any issues.

**We clarify that the dataset for the research described in this journal is available open access from the University’s public repository SHURDA. We use the following words for the re-submission: “The dataset (Haake et al. parkrun data Plos Global Public Health 2021.xlsx) is available from the University’s public repository SHURDA) at** [**https://shurda.shu.ac.uk/cgi/users/home?screen=EPrint::View&eprintid=144#t**](https://shurda.shu.ac.uk/cgi/users/home?screen=EPrint::View&eprintid=144#t)**”**

**(Apologies for confusing the issue: the *full* survey (i.e. other data over and above that used in this paper) are available through the parkrun Research Board for secondary data analysis through a data sharing agreement: we welcome further analysis of this rich data set.)**

5. Please ensure you include your funder's role statement "The funders had no role in study design, data collection and analysis, decision to publish, or preparation of the manuscript." at the end of your amended statement

**The statement has been added.**

*Please review your reference list to ensure that it is complete and correct. If you have cited papers that have been retracted, please include the rationale for doing so in the manuscript text, or remove these references and replace them with relevant current references. Any changes to the reference list should be mentioned in the rebuttal letter that accompanies your revised manuscript. If you need to cite a retracted article, indicate the article’s retracted status in the References list and also include a citation and full reference for the retraction notice.*

**The reference list has been checked and is complete and correct.**

*[Note: HTML markup is below. Please do not edit.]

Reviewers' comments:

Reviewer's Responses to Questions*

***Comments to the Author*** *1. Does this manuscript meet PLOS Global Public Health’s* [*publication criteria*](https://journals.plos.org/globalpublichealth/s/criteria-for-publication)*? Is the manuscript technically sound, and do the data support the conclusions? The manuscript must describe methodologically and ethically rigorous research with conclusions that are appropriately drawn based on the data presented.*

*Reviewer #1: Yes*

*Reviewer #2: Yes*

*2. Has the statistical analysis been performed appropriately and rigorously?*

*Reviewer #1: Yes*

*Reviewer #2: Yes*

*3. Have the authors made all data underlying the findings in their manuscript fully available (please refer to the Data Availability Statement at the start of the manuscript PDF file)?

The* [*PLOS Data policy*](https://journals.plos.org/globalpublichealth/s/data-availability) *requires authors to make all data underlying the findings described in their manuscript fully available without restriction, with rare exception. The data should be provided as part of the manuscript or its supporting information, or deposited to a public repository. For example, in addition to summary statistics, the data points behind means, medians and variance measures should be available. If there are restrictions on publicly sharing data—e.g. participant privacy or use of data from a third party—those must be specified.*

*Reviewer #1: Yes*

*Reviewer #2: Yes*

*4. Is the manuscript presented in an intelligible fashion and written in standard English?

PLOS Global Public Health does not copyedit accepted manuscripts, so the language in submitted articles must be clear, correct, and unambiguous. Any typographical or grammatical errors should be corrected at revision, so please note any specific errors here.*

*Reviewer #1: Yes*

*Reviewer #2: Yes*

*5. Review Comments to the Author

Please use the space provided to explain your answers to the questions above. You may also include additional comments for the author, including concerns about dual publication, research ethics, or publication ethics. (Please upload your review as an attachment if it exceeds 20,000 characters)*

*Reviewer #1: Very well written submission. The manuscript contained a very detailed methodology and thorough explanation of the implementation which was appropriate to fully meet the overall objectives of the study. The large data set seems to have been rigorously analysed and support the conclusions. The majority of the results were presented in a clear and easily understandable format. However, figure 2 "Perceived impact of volunteering at parkrun" seems to be missing which should be amended.*

**Thank you for your kind review. Figure 2 was in our version of the pdf submitted and is also in the resubmission. We can only deduce that this was due to software incompatability between computers. We will work with the editor in case there is a problem with our file.**

*Reviewer #2: The manuscript is nicely written by the authors. It is very interesting work on the volunteers.
The methods used by the authors are appropriate and can be replicated easily. The analysis and interpretation is ok and discussed in multiple angles.*

**Thank you for your kind review.**

*6. PLOS authors have the option to publish the peer review history of their article (*[*what does this mean?*](https://journals.plos.org/globalpublichealth/s/editorial-and-peer-review-process#loc-peer-review-history)*). If published, this will include your full peer review and any attached files.*


***Do you want your identity to be public for this peer review?*** *If you choose “no”, your identity will remain anonymous but your review may still be made public.


For information about this choice, including consent withdrawal, please see our* [*Privacy Policy*](https://www.plos.org/privacy-policy)*.*

*Reviewer #1: No*

*Reviewer #2:* ***Yes:*** *Palash Chandra Banik*
